# Ultrasound Guided Maxillary Nerve Block for Perioperative Pain Management for Patients Undergoing Endoscopic Sinus Surgery: Randomized Control Trial

**DOI:** 10.5812/aapm-144074

**Published:** 2024-04-03

**Authors:** Mahmoud Badry Ahmed, Ahmed Zaghloul, Ahmed Maarouf, Mohammed M Maarouf, Minatallah Elshafie

**Affiliations:** 1 Kasr Alainy, Cairo University, Giza, Egypt; 2 Ain Shams University, Cairo, Egypt; 3 Menofia University, Al Minufiyah, Egypt

**Keywords:** Functional Endoscopic Sinus Surgery, Maxillary Nerve Block, Ultrasound Guidance, Perioperative Analgesia

## Abstract

**Background:**

Proper perioperative pain management remains a cornerstone of well-conducted functional endoscopic sinus surgery (FESS). In such a context, proper pain management entails the adequate provision of prolonged postoperative analgesia, the avoidance of overusing opioids, and consequently limiting their unwanted side effects.

**Objectives:**

We aimed to evaluate the effect of bilateral ultrasound-guided suprazygomatic maxillary nerve block (MNB) on postoperative pain in patients undergoing FESS.

**Methods:**

Patients eligible for FESS were randomized into two groups: The MNB group (n = 30), who underwent bilateral ultrasound-guided suprazygomatic maxillary nerve block after induction of anesthesia, and a control group (n = 30), who received multimodal analgesia, including opioids. Postoperatively, patients were observed for 48 hours. Pain scores were evaluated upon arrival to the sPACU and at 2, 6, 12, 24, 36, and 48 hours postoperatively, particularly at the time of removal of the hemostatic agent after 36 hours postoperatively. Total rescue analgesia, postoperative complications (including nausea and vomiting [PONV], hypotension, bradycardia, headache), and patient satisfaction were also diligently recorded.

**Results:**

Sixty patients who were candidates and underwent FESS surgery were enrolled randomly in both groups. The NRS pain score in the MNB group was significantly lower than that of the control group (P < 0.001), especially the NRS during the removal of the hemostatic agent at T10 was significantly lower in the MNB group (P < 0.001). However, at the 24 - hour point post-surgery, there were no significant differences between both groups (P = 0.568). Total rescue analgesia required was significantly lower in the MNB group compared with the control group (P < 0.001) throughout the first 48 hours postoperatively. The percentage of patients with no postoperative complications (nausea, vomiting, and headache) was higher in the MNB group (76.7 %) compared with the control group (40 %). Patient satisfaction was statistically significantly higher in the MNB group (P < 0.001).

**Conclusions:**

Bilateral ultrasound-guided suprazygomatic MNB appeared to be safe and advantageous, as its use was associated with a decrease in total analgesic consumption, a reduction in postoperative morbidities such as pain, nausea, and vomiting, and greater patient satisfaction.

## 1. Background

Functional endoscopic sinus surgery (FESS) is currently the treatment of choice for conditions such as nasal polyps, chronic inflammatory paranasal sinus disease, and chronic rhinosinusitis (1). Image-guided surgery has improved patient safety and led to an increase in the number of FESS procedures performed for the treatment of nasal conditions.

Anesthesiologists encounter several challenges during FESS procedures. Proper airway and pain management during FESS should not hinder surgical access and should ensure safe emergence from anesthesia without associated bucking or straining that may lead to laryngospasm. Additionally, anesthesiologists should aim to minimize post-extubation hypertensive responses and resultant profuse microvascular bleeding. Prompt management of postoperative pain in patients undergoing FESS is necessary to minimize discomfort (2) and associated anxiety, ensure patient satisfaction, and reduce the need for opioid analgesics (3). Importantly, patients undergoing FESS are typically at risk for unanticipated overnight hospital admission and early hospital readmission due to nasal bleeding, pain, or intolerance to nasal packing or dressings (4).

Multimodal analgesia with maxillary nerve block and the use of alternatives to opioids can help minimize opioid use and provide effective pain control (5). Regional anesthesia for maxillofacial surgery has been reported to reduce intraoperative stress responses and perioperative opioid consumption, thus constituting a better and safer anesthesia technique (6).

Maxillary nerve block (MNB) was first described in the early 20th century as a method of anesthesia for dental purposes (7). Its use has recently expanded to anesthesia during surgeries such as cleft palate surgery, oral and orthognathic surgery, and repair of maxillary bone fractures. The ultrasound-guided suprazygomatic approach at the level of the suprazygomatic angle has been proposed as the safest, easiest, and most reliable approach to the pterygopalatine fossa (PPF) and achieving a successful MNB (8). This approach avoids penetration of the base of the skull and the orbit. Bony landmarks used are more superficial and more easily palpated, simplifying block performance and improving safety (9).

## 2. Objectives

We aimed to evaluate the efficacy and safety of the ultrasound-guided maxillary nerve block technique in perioperative pain management for patients undergoing endoscopic sinus surgery. Evaluation involved diligently recording any required rescue analgesia, keeping a record of the Numeric Rating Scale (NRS) pain scale, noting any postoperative complications, and assessing patient satisfaction.

## 3. Methods

### 3.1. Patients

This comparative study included 60 patients scheduled to undergo endoscopic sinus surgery. Approval was obtained from the local research ethics committee of the Faculty of Medicine, Ain Shams University, Egypt (FMASU R 97/2022). This trial was prospectively registered with the Clinical Trial Registry (PACTR202206548559545) on June 15, 2022, initiated on June 20, 2022, and concluded on November 15, 2022.

After obtaining written informed consent, patients were allocated to one of two groups using a random number generator in sealed envelopes. The first group (MNB group) was scheduled to undergo ultrasound-guided maxillary nerve block for pain management (n = 30). The other group (control group) was designated to receive multimodal analgesia with the use of opiates (n = 30).

Patients aged 20 - 60 years of both genders with American Society of Anesthesiologists (ASA) physical statuses of I or II who were candidates for FESS (for the correction of refractory, resistant chronic rhinosinusitis and/or polyps) were included in the study. Exclusion criteria included uncontrolled hypertension, cardiovascular or cerebrovascular disease, and chronic renal disease. Additionally, patients with a history of allergy to local anesthetics, opioid consumption, or those unwilling to provide informed consent were excluded from the study.

Upon admission to the operating room, standard basic anesthesia monitoring was applied to patients. After pre-oxygenation using an O2/Air mixture (FiO2 = 0.8) for 3 - 5 min, general anesthesia was induced with intravenous 2 - 2.5mg/kg of propofol, 2µg/kg of fentanyl, and 0.6 mg/kg rocuronium, followed by tracheal intubation.

Patients were maintained on isoflurane in oxygen, and mechanical ventilation was adjusted to keep SaO2 > 95 % and end-tidal CO2 between 35 - 45 mmHg. One gram of paracetamol infusion was administered as part of the analgesia. All patients received 8 mg of dexamethasone as prophylaxis against airway edema, as well as 8 mg of ondansetron as an antiemetic. For the operation, patients were positioned in the reverse Trendelenburg position at an angle of 15˚. A decongestant in the form of epinephrine 1:200,000 was administered by the surgeon into the nasal cavity.

**Figure 1.**
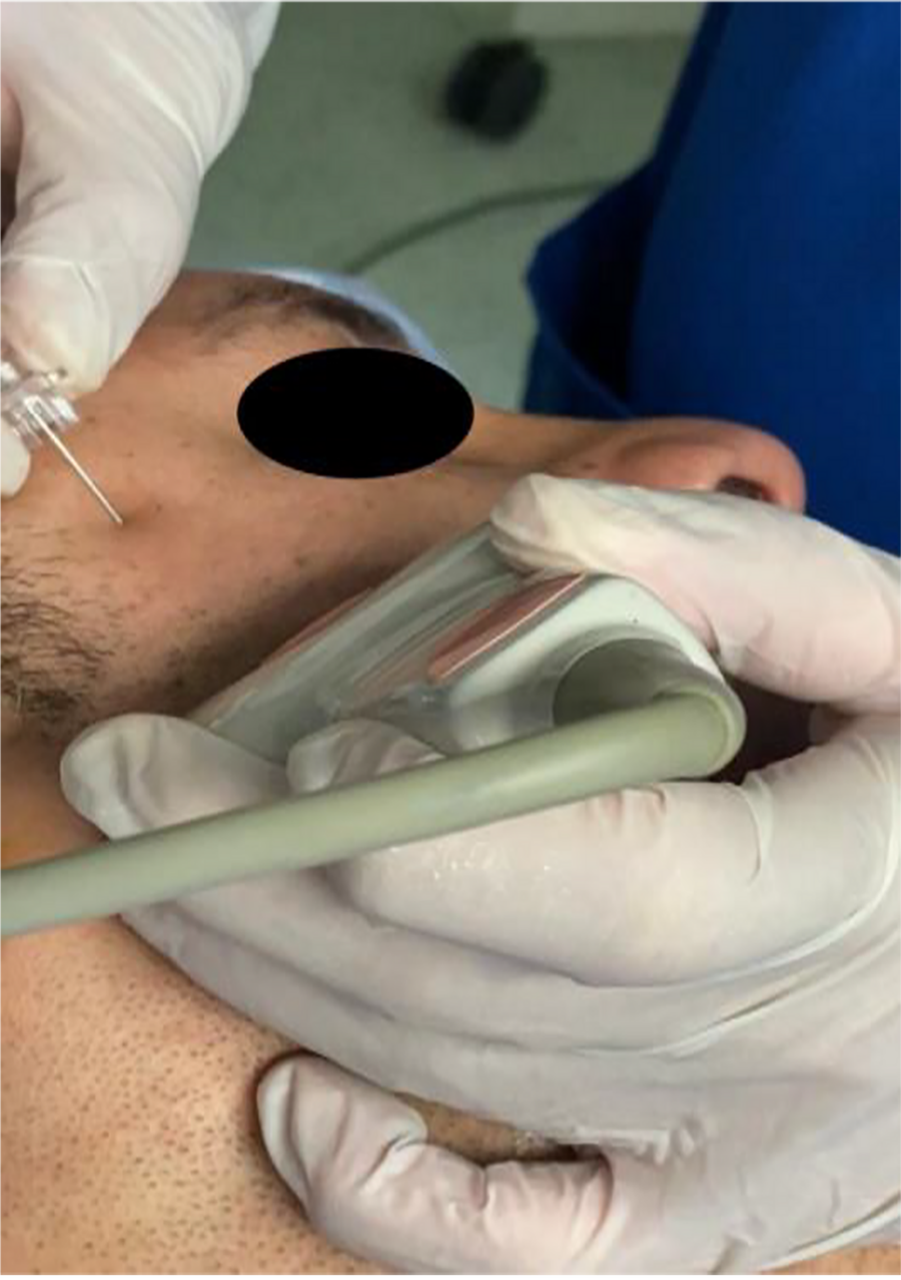
The ultrasound probe in the infrazygomatic area, with an inclination of 45 and the needle at the frontozygomatic angle and redirected to the PPF

**Figure 2.**
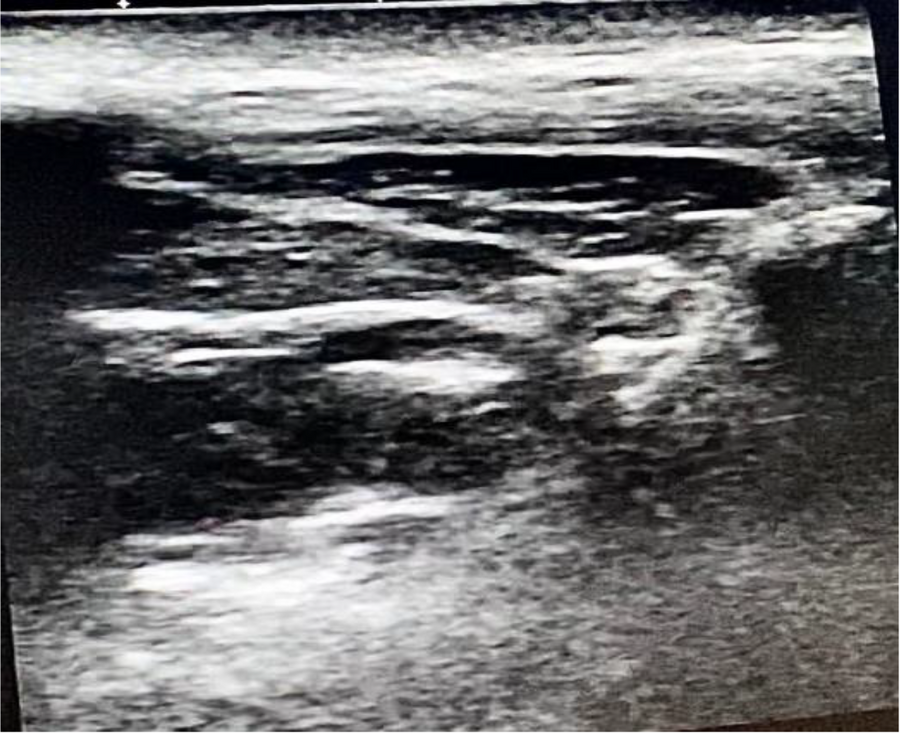
Ultrasound imaging for the PPF after injection of the local anaesthetics

Following induction of anesthesia, maxillary nerve block was administered to patients in the MNB group by the most experienced anesthetist present. This was followed by the skin incision. The skin was disinfected with 2 % chlorhexidine in 70 % alcohol, and an 8 to 13 - MHz linear-array ultrasound transducer (TOSHIBA, Model USAP-770A, JAPAN) was placed in the infrazygomatic area, (10) with an inclination of 45˚ in the transverse plane as shown in Figure 1. A flange of a 20-gauge needle was inserted perpendicular to the skin at the frontozygomatic angle (bounded by the superior edge of the zygomatic arch below and the posterior orbital edge forward), and advanced to the greater wing of the sphenoid. The needle was then redirected and advanced to the pterygopalatine fossa (PPF). Subsequently, aspiration was conducted, and then local anesthetic in the form of 1.5 mL of 0.5 % bupivacaine with an added adjuvant of 1 mL of dexamethasone (4 mg) in PPF was administered. This combination was deemed sufficient to be efficacious and safe during the block procedure (8) (see Figure 2). 

At the end of surgery, patients were extubated and transferred to the post-anesthesia care unit (PACU). Discharge was based on the Post-Anesthesia Discharge Scoring criteria (11).

Postoperative pain management for all patients started with the immediate infusion of 1 gm of paracetamol. Paracetamol infusion was readministered, if needed, every 8 hours. For the control group, 30 mg of intravenous ketorolac was administered and readministered, if needed, after 12 hours.

Postoperative pain was evaluated in both groups using the NRS pain score. Patients were asked to choose a number between 0 and 10 that best reflected the intensity of their pain, where zero represented 'no pain at all' and 10 represented 'the worst pain ever experienced'. Any patient with an NRS score of 4 or more received 10mg/70kg of nalbuphine, administered intravenously as rescue analgesia.

The hemostatic agent introduced at the end of surgery was removed 36 hours after the operation. Removal was understandably painful and, in most cases, required analgesia.

### 3.2. Measured Data

Demographic data, including age, gender, body mass index (BMI), and ASA status, were carefully recorded. Measurements of systemic hemodynamics, including patient heart rate and median blood pressure, were recorded throughout the surgery, upon admittance to the PACU, and at 2, 6, and 12 hours postoperatively. Results of pain assessment using the NRS pain score were recorded immediately after induction of anesthesia, upon admittance to the PACU, and at 2, 6, 12, 24, 36, and 48 hours postoperatively. The NRS pain score, as well as any required analgesia, were both recorded at the time of removal of the hemostatic agent. Total rescue analgesia and postoperative complications (headaches, nausea/vomiting, and bleeding) were diligently recorded during the first 48 hours. Lastly, patient satisfaction was assessed and carefully recorded. Patients responded to relevant questions by selecting responses ranging from 'Not satisfied' = 1, 'Less satisfied' = 2, 'Quite satisfied' = 3, 'Satisfied' = 4, to 'Very satisfied' = 5.

The primary goal of the study was to evaluate the efficacy of MNB for FESS by assessing pain at admittance to the PACU and at 2, 6, 12, 24, 36, and 48 hours postoperatively, and comparing pain scores during the removal of the hemostatic agent in the two groups. Our secondary goals were to assess each of the following: Total rescue analgesia required; postoperative complications during the first 48 hours; and patient satisfaction.

### 3.3. Statistical Analysis

Data were analyzed using the Statistical Package for Social Science (IBM Corp, released 2013. IBM SPSS Statistics for Windows, V. 22.0. Armonk, NY, USA). Parametric quantitative data were expressed as mean ± standard deviation (SD), while non-parametric quantitative data were expressed as median and IQR. Qualitative categorical variables were analyzed as frequency and percentage. The Chi-square test was used to evaluate differences between categorical data. The independent sample T-test was utilized to assess differences between normally distributed independent parametric quantitative variables. The Mann-Whitney U Test was employed to evaluate differences between ordinal variables and independent non-parametric quantitative variables that were not normally distributed. All P values were two-tailed, and a P-value < 0.05 was considered statistically significant.

## 4. Results

**Figure 3.**
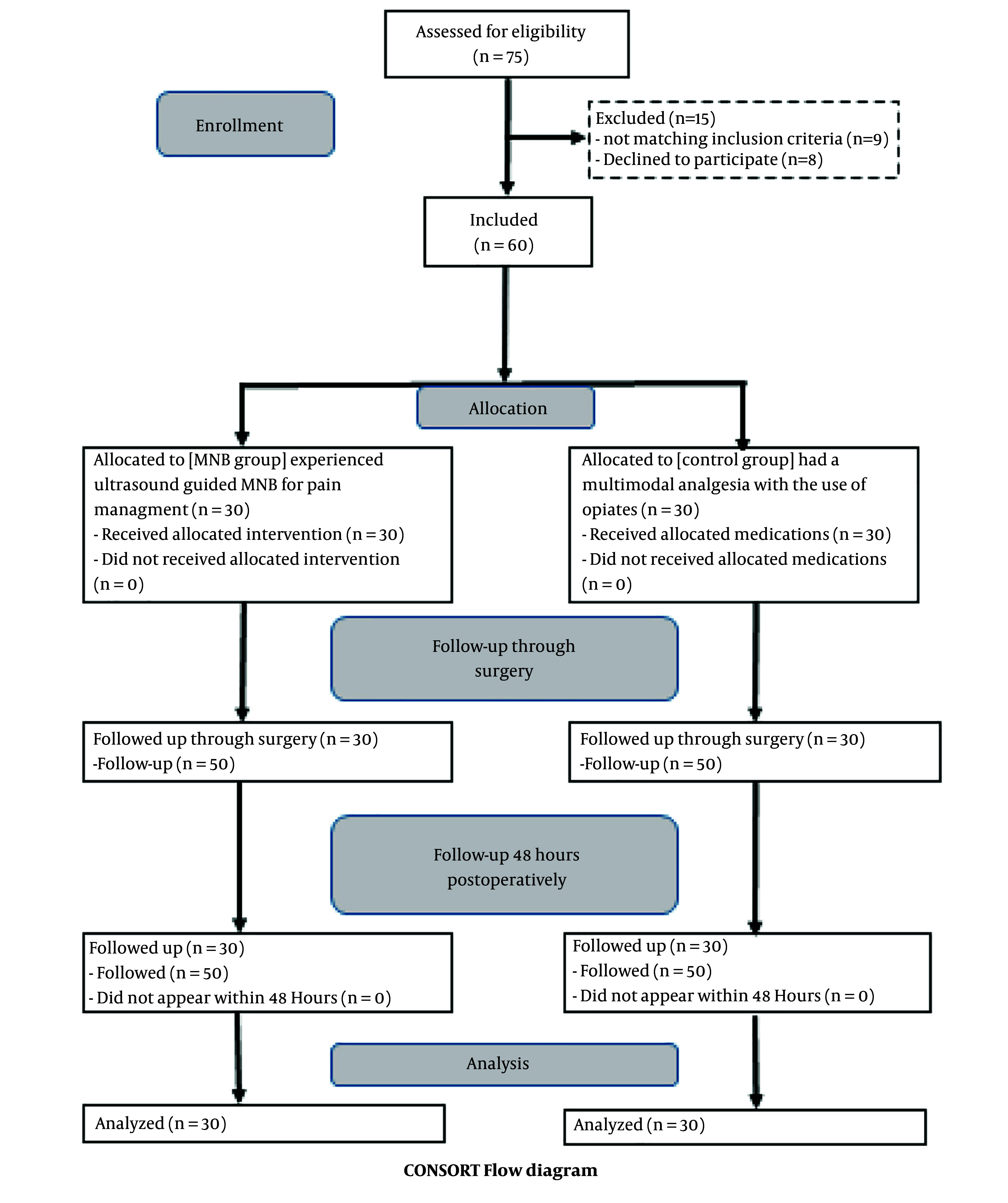
Consort flow diagram

Sixty patients were enrolled in this randomized, double-blinded study and subsequently underwent FESS surgery, as depicted in the consort flow diagram (Figure 3). Both groups showed no significant differences regarding demographic data or duration of surgery ([Table A144074TBL1]). 

**Table 1. A144074TBL1:** Demographic Data, Time of Surgery, Postoperative Complications and Total Rescue Analgesia Among Both Groups ^a^

Variables	Control	MNB	Test	P-Value
**Age (range)**	31.57 ± 7.749 (20 - 46)	30.13 ± 6.399 (21 - 43)	0.781^b^	0.438
**BMI (range)**	31.77 ± 2.661 (28 - 37)	31.83 ± 2.547 (28 - 36)	-0.099 ^b^	0.921
**Sex **			0.067 ^c^	1
Male	15 (50.0)	16 (53.3)		
Female	15 (50.0)	14 (46.7)		
**ASA**			1.071 ^b^	0.438
1	14 (46.7)	18 (60.0)		
2	16 (53.3)	12 (40.0)		
**Postop Complications**			10.900 ^d^	0.015
No	12 (40.0)	23 (76.7)		
Bleeding	2 (6.7)	3 (10.0)		
Headache	6 (20.0)	2 (6.7)		
Nausea	9 (30.0)	2 (6.7)		
Nausea and vomiting	1 (3.3)	0 (0.0)		
**Total rescue Analgesia**	25.67 (30)	11.33 (20)	-5.6494 ^e^	< 0.001
**Surgery time MIN**	91.13 (30)	90.67 (30)	-0.2664 ^e^	0.790

^a^ Values are expressed as No. (%) or mean ± SD.

^b^ Independent *t*-test.

^c^ Chi-square tests.

^d^ Fisher's exact test.

^e^Mann-Whitney U.

The NRS score was recorded immediately after recovery from anesthesia (upon admittance to the PACU), as well as after 2, 6, 12, 24, 36, and 48 hours. The NRS in the MNB group was significantly lower than that of the control group (P < 0.001). However, there were no significant differences between the groups with regards to the NRS score only at the 24-hour point post-surgery (P = 0.568). The NRS pain score during the removal of the hemostatic agent at T10 was significantly lower in the MNB group (median = 5.0) compared with the control group (median = 7.0) (P < 0.001) (Table 2). 

**Table 2:** . Serial NRS Score Among Both Groups ^a^

Variable	Median	Min	Max	Range	IQR	Test	P-Value
**T5 NRS**							< 0.001
Control	5.00	4	8	4	2	-6.707	
MNB	2.50	2	4	2	1		
**T6 NRS**							< 0.001
Control	4.00	4	6	2	1	-6.074	
MNB	3.00	2	4	2	0		
**T7 NRS**							< 0.001
Control	4.00	4	7	3	1	-5.137	
MNB	3.50	2	4	2	1		
**T8 NRS**							0.008
Control	4.00	4	6	2	0	-2.659	
MNB	4.00	3	6	3	1		
**T9 NRS**							0.568
Control	4.00	4	4	0	0	-0.571	
MNB	4.00	3	6	3	1		
**T10 NRS**							< 0.001
Control	7.00	5	10	5	2	-4.288	
MNB	5.00	4	8	4	1		
**T11 NRS**							< 0.001
Control	4.00	4	4	0	0	-7.307	
MNB	2.00	2	3	1	1		

^a^Test; Mann-Whitney U.

Total rescue analgesia required was significantly lower in the MNB group (median = 11.33) compared with the control group (median = 25.67) throughout the first 48 hours postoperatively (Table 1). 

**Figure 4.**
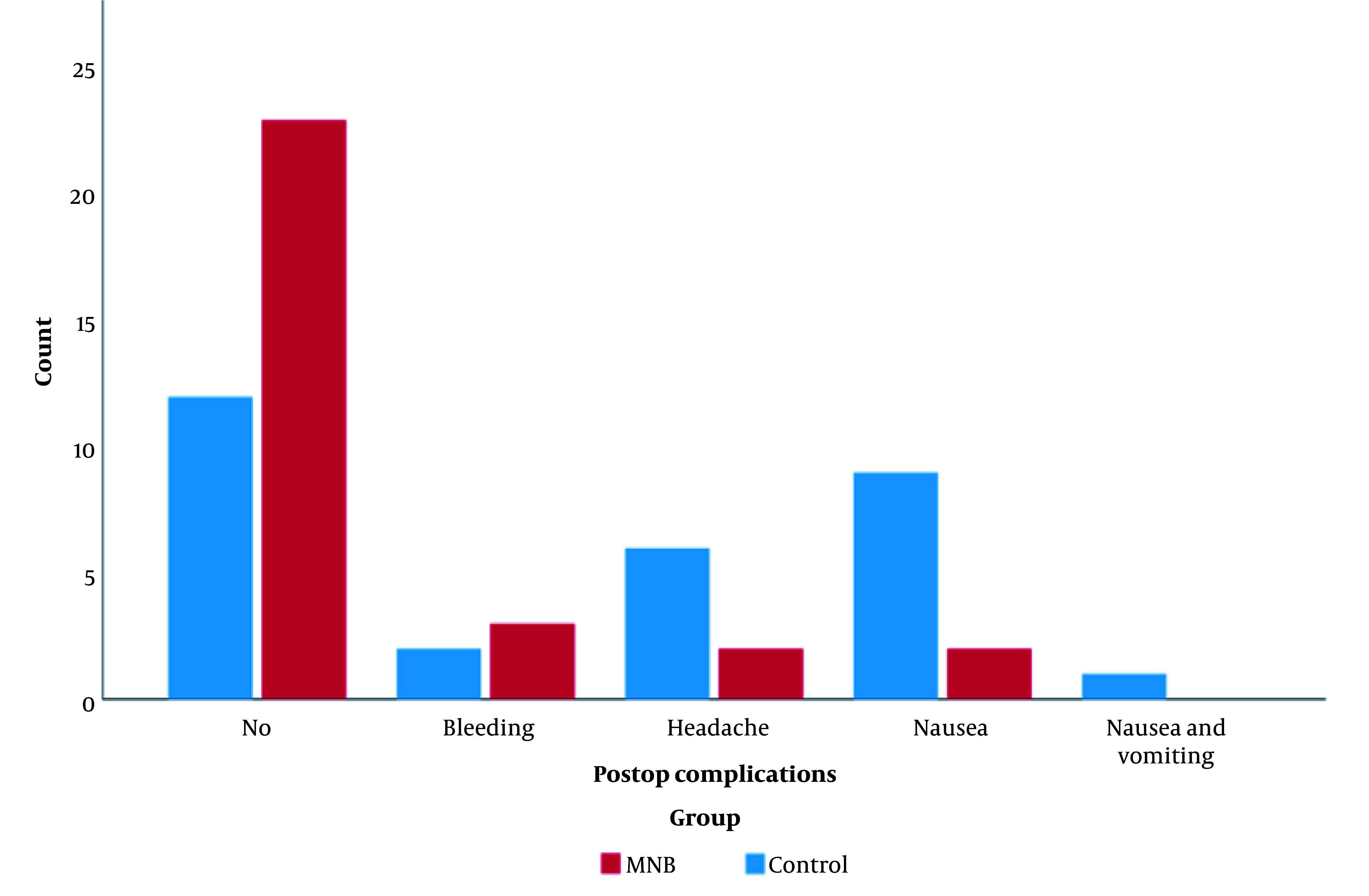
Clustered column chart showing the distribution of postop complications in the control and MNB groups. There is a statistically significant difference between the post-operative distribution in the two groups, while nausea (30%) was the most frequent post-operative in the control group, bleeding (10%) was the most frequent postoperative in the MNB group. Additionally, there were fewer postoperative complications in the MNB (76.7%) group than in the control (40%) group.

There was a statistically significant difference between the distribution of postoperative complications in the two groups. While nausea was the most frequent postoperative complication in the control group (9 patients (30.0 %)) versus the MNB group (2 patients (6.7 %)), vomiting was only reported in the control group (1 patient (3.3 %)). Headaches were more frequent among patients of the control group (6 (20.0 %)) compared with the MNB group (2 patients (6.7 %)). Additionally, a significantly larger number of patients in the MNB group experienced no postoperative complications (76.7 %) compared with the control group (40 %). No serious complications such as infection and ocular lesions were reported in either group (Table 1, Figure 4). 

**Figure 5.**
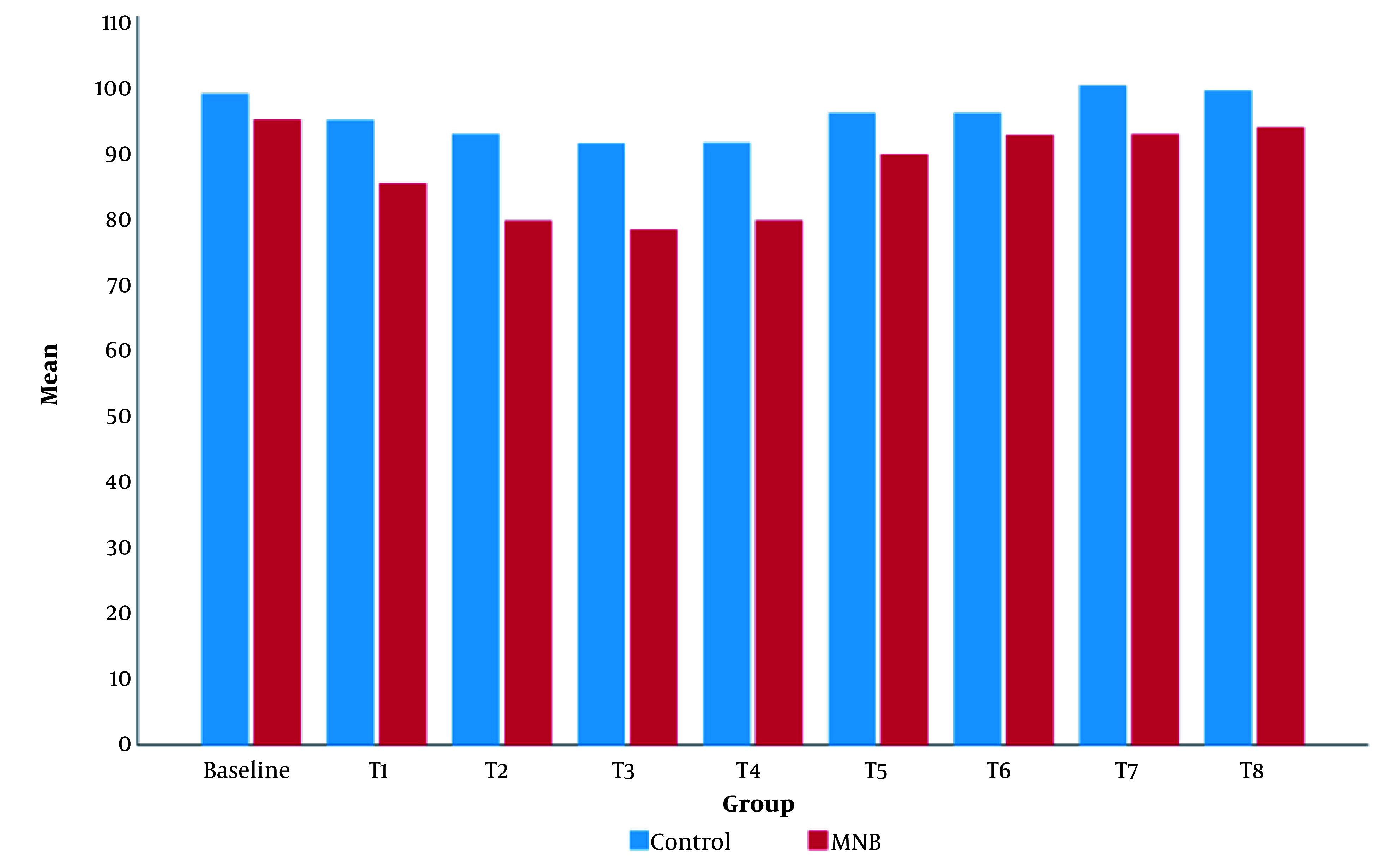
Clustered column chart showing the mean of serial MBP in the control and MNB groups. The MNB group's mean value of all MBP was significantly lower than that in the control group.

**Figure 6.**
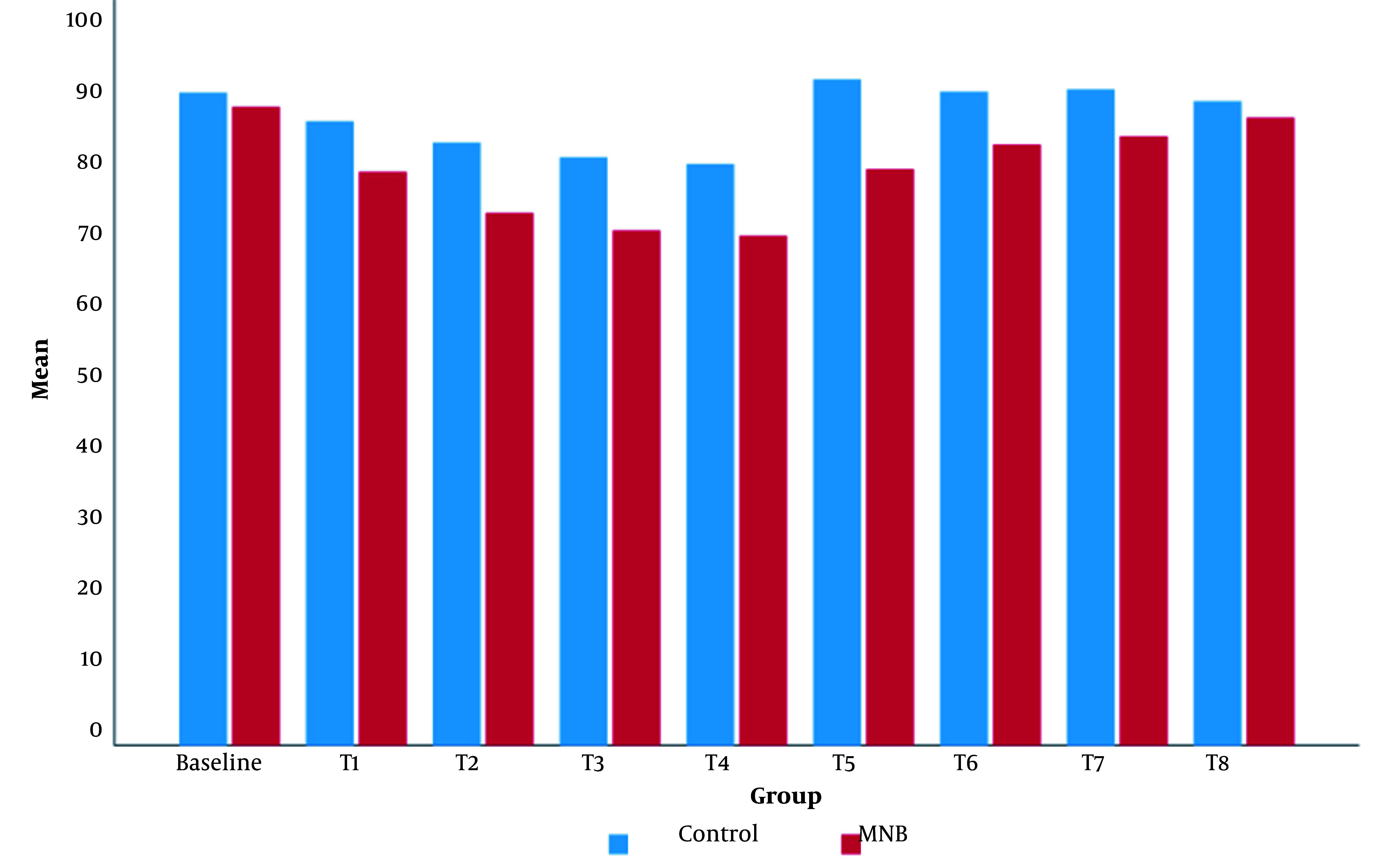
Clustered column chart showing the mean of serial HR in the control and MNB groups. The mean value of all HR in the MNB group was significantly lower than that of the control group, except for the T8, where there was no statistically significant difference between the two groups.

Intraoperatively, the mean value of MBP was significantly lower in the MNB group than in the control group (Figure 5). The mean value of HR in the MNB group was significantly lower than that of the control group, except for at T8 (12 hours after the surgery), where there was no statistically significant difference between the two groups (Figure 6). 

Patient satisfaction was statistically significantly higher in the MNB group, with 56.7 % of MNB group patients choosing ‘very satisfied’ versus only 13.3 % of those in the control group (p < 0.001) (Table 3). 

**Table 3.** Patient Satisfaction Among Both Groups ^a^

Variables	Control	MNB	Test ^b^	P-Value	Test ^c^	P-Value
**Not satisfied**					-3.595	< 0.001
Count	2	0	0	1		
% Within patient satisfaction	100.0	0.0				
% Within group	6.7	0.0				
**Less satisfied**						
Count	3	2	0.200	0.655		
% Within patient satisfaction	60.0	40.0				
% Within group	10.0	6.7				
**Quite satisfied**	13	4	4.765	0.029 ^c^		
Count						
% Within patient satisfaction	76.5	23.5				
% Within group	43.3	13.3				
**Satisfied**						
Count	8	7	0.067	0.796		
% Within patient satisfaction	53.3	46.7				
% Within group	26.7	23.3				
**Very satisfied**						
Count	4	17	-4.472	< 0.001 ^c^		
% Within patient satisfaction	19.0	81.0				
% Within group	13.3	56.7				

^a^Values are expressed as No. (%).

^b^ Chi square test.

^c^ Significant.

## 5. Discussion

This study has demonstrated the efficiency of ultrasound-guided suprazygomatic maxillary nerve block in the management of postoperative pain after FESS operation during the first 48 hours postoperatively. Our results showed lower NRS scores in the MNB group compared to the control group, resulting in a smaller amount of rescue analgesia required with a lower incidence of postoperative complications.

Comparable results were reported by Rezaeian et al. (12), who studied the effect of sphenopalatine ganglion block (SPGB) with bupivacaine on postoperative pain in 40 patients undergoing FESS surgery. They concluded that SPGB with bupivacaine 0.5 % (1.5 mL) was an effective and non-invasive method of postoperative pain management. In our research, the block was performed under ultrasound guidance, allowing for the visualization of vascular structures and the avoidance of accidental puncture during the block procedure (13).

Another study by Al-Qudah (5) evaluated the efficacy of bilateral endoscopic injection of lidocaine with epinephrine in the sphenopalatine ganglion at the end of FESS on postoperative pain and requirement for rescue analgesia. They reported that it was an effective method of short-term pain control. DeMaria et al. (14) studied the use of bilateral SPG in adults scheduled for sinus surgery. They used 1 mL of 1% lidocaine with epinephrine during the procedure, with oxymetazoline nasal spray administered 30 minutes before the surgery. They deduced that this combination had shortened the hospital stay and reduced requirements for narcotics, but they noted no additional benefits with regards to pain management beyond 24 hours postoperatively.

Comparatively, our results showed a significant reduction in NRS scores at 36 hours post-surgery, which coincided with the time of removal of the haemostatic agent (described as the most unbearable pain ever by most of the patients), as we used 1 ml dexamethasone as an adjuvant with an additional 1.5 mL of 0.5 % bupivacaine in the MNB. Similarly, Mansour RF and Abdelghany (15) showed that administering 0.5 µg/kg dexmedetomidine as an adjunct to bupivacaine 0.25 % in MNB to children assigned for the surgical correction of cleft palates resulted in prolonged postoperative analgesia and decreased total analgesic consumption.

In this study, there was a significant reduction in the incidence of postoperative complications in the MNB group, accompanied by better patient satisfaction. Comparable results were reported by Abubaker AK and Al-Qudah., who concluded that injecting the SPG with a local anesthetic at the end of surgery significantly reduced the incidence of PONV after FESS (16). Additionally, a meta-analysis by Kim et al., conducted on a number of studies that included a total of 441 participants, concluded that SPGB administered after FESS had provided effective control of postoperative morbidities such as pain and nausea and vomiting (17).

On the contrary, Cho et al. (18) found no statistical differences in daily pain medication requirements (both acetaminophen and opioid) following FESS between their two patient groups across all time points, and the number of patients who did not require any pain medications was also similar in the two groups. It is important to note that they used a lower concentration of bupivacaine 0.25% with epinephrine in the treatment group, while we administered a higher concentration of 0.5% bupivacaine with dexamethasone as an adjuvant.

We observed no major intra or postoperative complications in either group, including any hemodynamic instability affecting either patient's arterial blood pressure or heart rate. The lack of observable major complications was consistent with the results of Rezaeian et al. (12), who recorded the hemodynamic data of the patients in the operating room and in the recovery room and found no significant differences between the groups.

One limitation of this study was its single-center nature. Another limitation was our use of only a small dose of dexamethasone as an adjuvant to the local anesthetic. Further studies are thus required to determine the efficacy of other possible adjuvants or higher doses of adjuvants used in this study on the duration and quality of pain management post-sinus surgery while employing the same block technique described by this study.

### 5.1. Conclusions

This study demonstrated that bilateral ultrasound-guided suprazygomatic maxillary nerve block was safe and efficacious, as it decreased total analgesic consumption. It contributed to improved patient outcomes by providing effective control of postoperative morbidities such as pain, nausea, and vomiting, and was associated with higher patient satisfaction with surgery among patients assigned for FESS.

## Data Availability

The dataset presented in the study is available on request from the corresponding author during submission or after publication.

## Clinical Trial Registration Code:

PACTR202206548559545.
